# Attitudes of Primary School Teachers Toward Inclusive Education

**DOI:** 10.3389/fpsyg.2022.891930

**Published:** 2022-05-06

**Authors:** Jasmina Radojlovic, Tatjana Kilibarda, Svetlana Radevic, Milena Maricic, Katarina Parezanovic Ilic, Milan Djordjic, Sofija Colovic, Branimir Radmanovic, Marija Sekulic, Ognjen Djordjevic, Jovan Niciforovic, Ivana Simic Vukomanovic, Katarina Janicijevic, Snezana Radovanovic

**Affiliations:** ^1^College of Health Studies Milutin Milankovic, Belgrade, Serbia; ^2^Department in Cuprija, The Academy of Applied Preschool Teaching and Health Studies Krusevac, Cuprija, Serbia; ^3^Department of Social Medicine, Faculty of Medical Sciences, University of Kragujevac, Kragujevac, Serbia; ^4^Department School of Applied Health Science Studies, Academy of Applied Studies Belgrade, Belgrade, Serbia; ^5^Department of Physical Medicine and Rehabilitation, Faculty of Medical Sciences, University of Kragujevac, Kragujevac, Serbia; ^6^Department of Communication Skills, Ethics and Psychology, Faculty of Medical Sciences, University of Kragujevac, Kragujevac, Serbia; ^7^Department of Psychiatry, Faculty of Medical Sciences, University of Kragujevac, Kragujevac, Serbia; ^8^Department of Hygiene and Ecology, Faculty of Medical Sciences, University of Kragujevac, Kragujevac, Serbia; ^9^Department of Epidemiology, Faculty of Medical Sciences, University of Kragujevac, Kragujevac, Serbia; ^10^Department of Natural Sciences, Faculty of Hotel Management and Tourism in Vrnjacka Banja, University of Kragujevac, Vrnjacka Banja, Serbia

**Keywords:** inclusive education, primary school teachers, attitudes, children with disabilities, quality and education

## Abstract

**Background:**

The aims of our study are related to examining the relevance of teachers' attitudes toward the implementation of inclusive education. In addition, its subject is related to the implications on inclusive education policies, limitations of the existing study along with the recommendations for our future research endeavors.

**Methods:**

The research is a cross-sectional study type. The sample included 64 primary school teachers in the lower grades of primary school (grades 1–4), selected by using simple random sampling, in three primary schools on the territory of Belgrade, Serbia in 2021 (26, 17, and 21 primary school teachers). The Questionnaire for Teachers, which was used as a research instrument, was taken from the Master's Thesis Studen Rajke, which was part of the project “Education for the Knowledge Society” at the Institute for Educational Research in Belgrade. Dependent variables measured in the study referred to the attitudes of primary school teachers toward inclusive education. Categorical variables are represented as frequencies and the Chi-square test was used to determine if a distribution of observed frequencies differed from the expected frequencies.

**Results:**

One in three teachers (32.8%) thought that inclusion was useful for children with disabilities (29.7%), of them thought that schools did not have the conditions for inclusive education, whereas one in four teachers (25.0%) believed that inclusion was not good. No statistically significant differences were found in the attitudes of professors, when observed in terms of their gender, age and length of service.

**Conclusion:**

Investing more resources and time in developing and implementing special education policies can promote successful inclusive education.

## Introduction

Inclusive education means quality education for all students, respecting their diversity in terms of educational needs (UNESCO, 2009). The inclusion of students with disabilities in typical schools with their peers is part of the global human rights movement, which refers to the possibility that students with disabilities can fully participate in all activities that make up modern society (Rajšli-Tokoš, 2020).

The history of inclusive education dates back since the *World Conference on Special Needs Education: Access and Quality* (Salamanka, Spain, 7–10 June 1944), which enabled substantial progress, simultaneously opening room for improvement and introducing a more dynamic approach of schools toward all children, irregardless of their physical, intellectual, social, emotional, language or other state of mind, as well as the educational system oriented toward the students with various educational needs, by means of which the inclusive education platform was eventually established (United Nations Educational, 1994). The necessity of implementing inclusive education was confirmed at the UNESCO World Conference on Special Needs Education (UNESCO, 2003). The principles of inclusive education were presented for the second time at the World Education Forum meeting in Dakar in the year of 2000 (The Dakar Framework for Action, 2000), and finally, they were confirmed in the Millennium Declaration (Bhaskara, 2003). A few years later, at the Second European Ministerial Conference, which took place in Malaga, Spain, the Council of Europe (Council of Europe, 2003) declared that education is the fundamental means enabling children with disabilities to successfully integrate into the community and it established the legal framework needed for including special needs children in regular schools by providing necessary support and thus promoting the ways in which their education could be improved (Council of Europe, 2006).

There is a large number of studies which indicated that inclusive education has its own advantages as regards cognitive, social and affective development of students. They also show that inclusive education, compared to segregated education, offers more opportunities to develop social, emotional, and behavioral skills not only of children who need additional support, but also of children of typical development in terms of their enhancing the level of understanding and acceptance of diversity (Magyar et al., 2020; Molina Roldán et al., 2021; van Kessel et al., 2021). The results of various research studies have shown that students without difficulties have positive attitudes, positive beliefs, and express readiness when it comes to accepting students with disabilities along with having a positive attitude toward joint teaching with them, which is a very important factor for successful inclusion (Radisavljevic-Janic et al., 2018; Alnahdi and Schwab, 2021).

Additionally, it was confirmed that teachers were the key actors in the implementation of inclusive education and that their positive attitudes played a significant role in the successful administration of this educational transformation (De Boer et al., 2011). However, whereas the effects of teachers' attitudes toward the application of inclusion policies have been widely recognized, there is not a significant number of studies examining the factors shaping such attitudes.

Only a few studies revealed the effects of factors (age, gender, length of service (years), training, experience with inclusive education and type of student disability) on the attitudes of teachers toward inclusive education (Avramidis and Norwich, 2002; Alghazo et al., 2003; Forlin et al., 2009; Woodcock, 2013).

In this context, our study's primary goal is to examine attitudes of teachers toward inclusive education, sociodemographic factors affecting such attitudes along with the factors stated by teachers as being alleviating/aggravating factors for the inclusion process in education, which could be perceived as excellent basis for creating specialized programs and measures for the purpose of enhancing the inclusive education.

## Materials and Methods

### Study Design

The research is a cross-sectional study type.

### The Study Participants

The sample included 64 primary school teachers in the lower grades of primary school (grades 1–4), employed in three primary schools in the territory of Belgrade, Serbia, who were selected by using simple random sampling (26, 17, and 21 primary school teachers of both gender, with the average age of 43.1 ± 8.4 years). Data collection was realized in the second term of school year 2020/21, by means of a teacher survey. The school principals gave their consent to administering such teacher surveys or questionnaires.

### The Procedure for Data Acquisition

Teacher survey questionnaires were administered after delivering detailed written instructions which had been distributed in schools. Teacher survey questionnaires were voluntarily completed, and self-administered with respondents filling in their own answers. Before the start of the research, the respondents were acquainted with the goal and procedure of the research and gave their written consent to participate in the study. For the purpose of ensuring objectiveness of the results and capturing accurate data, during the procedure for data acquisition, researchers were leading teachers during the process of filling in their own answers in accordance with the methodically anticipated protocol. Before underlying the aims of the research and delivering instructions for the purpose of filling in the questionnaires given, the respondents were told that the data obtained in such a manner were going to be used for scientific purposes only, that all the answers were highly classified and that the analysis of data was going to be performed collectively, not individually. After placing an emphasis on the fact that filling in the questionnaires was anonymous, the researchers asked the respondents to be honest, to give answers to the questions independently, without sharing any mutual comments or chatting with other respondents. The respondents were given the manual for filling in the questions included in the questionnaire. The time expected to complete the questionnaire was 20 min ± several minutes.

### Instruments/Measures

The Questionnaire for Teachers, which was used as a research instrument, was taken from the Master's Thesis Studen Rajke (Studen, 2008), which was part of the project “Education for the Knowledge Society” at the Institute for Educational Research in Belgrade. The Teachers' Questionnaire examines the level of willingness that the primary school teachers have in order to accept children with disabilities, their experiences in working with children with disabilities, and their suggestions that would lead to more successful outcomes for children with disabilities. The questionnaire also contains a five-point scale consisting of 22 statements related to the importance of certain psychological and pedagogical measures undertaken for the purpose of successful implementation of inclusive education and factors that may be aggravating for the inclusive process. The independent variables examined in the research are sociodemographic characteristics of primary school teachers (such as gender, age, length of service). Dependent variables measured in the study referred to the attitudes of primary school teachers toward inclusive education (their readiness for inclusion, assessment of conditions for inclusion, difficulties, and benefits of inclusive education, providing support to children and parents involved in the inclusive education, the importance of psychological and pedagogical measures undertaken for the purpose of successful implementation of inclusive teaching, assessment of factors that may be aggravating for the inclusive process). The reliability of The Questionnaire for teachers in a sample of adult respondents was 0.86.

### Statistical Data Analysis

Statistical data processing was performed using SPSS software package, version 18.0. The results of the research are presented in Tables 1–4. Categorical variables are represented as frequencies and the Chi-square test was used to determine if a distribution of observed frequencies differed from the expected frequencies. A probability of <5% was considered statistically significant.

**Table 1:** Sociodemografic characteristics of primary school teachers and attitudes toward inclusive education.

**Variables**	**Total**	**Gender**		***p*** *	**Years of service**				***p*** *
		**Men**	**Women**		**Up to 5 years**	**5–9 years**	**10–14 years**	**15 and over**	
**Attitude toward inclusive education**								
Inclusive education requires a selective approach according to the type and severity of developmental pathology	60.9	25.0	18.2	0.765	20.0	52.6	0	0	<0.001
Inclusive education is an inalienable right of every child	20.3	60.0	61.4		73.3	42.1	90.9	0	
Inclusive education is a utopia	18.8	15.0	20.5		6.7	5.3	9.1	100.0	
**Experience in working with children with disabilities**								
Had experience with children with disabilities	31.3	50.0	22.7	0.069	20.0	52.6	27.3		0.138
Really rare	54.7	35.0	63.6		66.7	47.4	50.0		
They had no experience of this type	14.0	15.0	13.6		13.3	0	22.7		
**Conditions for inclusive education in the school**								
There are conditions in the school for inclusive education	20.3	25.0	18.2	0.530	13.3	42.1	13.6	0	0.034
Not there are conditions for inclusive education in the school	79.7	75.0	81.8		86.7	57.9	86.4	100.0	
**Difficulties in working with children with disabilities**								
Difficult adoption of materials	82.8	90.0	79.5	0.304	93.3	100.0	77.3	37.5	<0.001
Difficulty exercising emotional control	17.2	10.0	20.5		6.7	0	22.7	62.5	
**Benefits of inclusive education for children with disabilities**								
Inclusion useful for children with disabilities	32.8	40.0	29.5	0.676	33.3	63.2	18.2	0	<0.001
Schools do not have the conditions for inclusive education	42.2	40.0	43.2		60.0	31.6	50.0	12.5	
Iinclusion is not good	25.0	20.0	27.3		6.7	5.3	31.8	87.5	
**Is a regular or special school better for students with disabilities**								
Children with disabilities would be more successful in a special school	56.3	55.0	56.8	0.892	66.7	68.4	45.5	37.5	>0.263
Children with disabilities would be more successful in regular school	43.8	45.0	43.2		33.3	31.6	54.5	62.5	

* *Chi-square test*.

**Table 2:** Attitudes of primary school teachers toward children with disabilities.

**Offered questions**	**I totally agree**	**I partially agree**	**I'm not sure**	**I don't agree**	**I completely disagree**	***p*** *
Do you support the initiative to include children with disabilities in regular school, when it is possible?	7.8	57.8	18.8	18.8	1.6	<0.001
Does the inclusion of children with disabilities have a positive impact on all children in the group?	23.4	60.9	3.1	10.9	1.6	<0.001
In most respects, are children with disabilities equal to children without disabilities?	15.6	56.3	15.6	12.5	0	<0.001
The mutual education of children with disabilities and children of typical development is of mutual benefit	34.4	56.3	4.7	4.7	0	<0.001
A child with disabilities in the regular class of the Elementary School can negatively affect the success of the whole class	23.4	53.1	14.1	7.8	1.6	<0.001

* *Chi-square test*.

**Table 3:** Factors that would facilitate the process of inclusion in education.

**Offered answers**	**Very little**	**Little**	**Partly**	**Much**	**Very much**	***p*** *
Reducing excessive curricula	4.7	1.6	10.9	17.2	65.6	<0.001
Introduction of special programs to encourage the development of children with disabilities	1.6	0	9.4	24,9	64.1	<0.001
Adapting teaching contents to the abilities of these children - individual teaching	17.2	0	18.8	0	64.1	<0.001
Implementation of active, interactive and participatory methods	1.6	1.6	28.1	26.6	42.2	<0.001
Fewer students in the class	1.6	1.6	12.8	18.8	65.6	<0.001
Providing continuous professional assistance to the teacher by special educators	1.6	0	3.1	14.1	81.3	<0.001
Classes planned in such a manner so that all students can learn	3.1	0	18.8	35.9	42.2	<0.001
Encouraging the participation of all students in the class	1.6	6.3	28.1	26.6	37.5	<0.001
Developing students' understanding of diversity	1.6	6.3	14.1	18.8	59.4	<0.001
In every classroom where there are children with disabilities, there should be one special pedagogue in addition to the teacher	1.6	0	1.6	9.4	87.5	<0.001
Learning by working together in class	3.1	3.1	17.2	31.3	45.3	<0.001
Creating an individual educational plan for each child with disabilities	1.6	3.1	1.6	9.4	84.4	<0.001
Adequate adaptation of space	7.8	1.6	3.1	25.0	62.5	<0.001
Organizing seminars for teachers which enable them to work with children with disabilities	10.9	0	23.4	0	65.6	<0.001

* *Chi-square test*.

**Table 4:** Factors that complicate the process of inclusion in education.

**Offered answers**	**Very little**	**Little**	**Partly**	**Much**	**Very much**	***p*** *
Resistance of other children toward children with disabilities: ridicule, mockery, ignoring, etc.	12.5	4.7	14.1	26.6	42.2	<0.001
Resistance of parents of children of typical development toward inclusion	7.8	4.7	14.1	29.7	43.8	<0.001
Insufficient preparation of teachers to work with children with disabilities	1.6	3.1	14.1	32.8	48.4	<0.001
Insufficient motivation of teachers to accept additional obligations in their work	7.8	3.1	17.2	28.1	43.8	<0.001
The existing educational system, which is too difficult for other children	1.6	4.7	14.1	32.8	46.9	<0.001
Insufficient preparation of schools for accepting children with disabilities	1.6	1.6	9.4	45.3	42.2	<0.001
Lack of financial resources to implement fundamental school reform	1.6	0	10.9	21.9	65.6	<0.001

* *Chi-square test*.

## Results

As the figures indicate, males accounted for 31.3% of the total number of primary school teachers, whereas females accounted for 68.7%. The average age of respondents was 43.1 ± 8.4 years. The majority of respondents were aged between 40 and 49 years (39.4%), whereas the least number of respondents was in the group with an age of more than 60 years (3.9%).

A total of 23.4% of professors had a length of service of up to 5 years, 29.7% had a length of service from 5 to 9 years, a third had a length of service from 10 to 14 years, and 12.5% had a length of service of over 15 years. The largest percentage of them, 60.9%, believe that inclusive education requires a selective approach according to the type and severity of developmental pathology, 20.3% of them believe that it is an inalienable right of every child, whereas 18.8% believe that it is a utopia. Almost a third of professors (31.3%) stated that in their previous pedagogical work, they had experience with children with disabilities, while slightly more than half of them (54.7%) rarely had this particular experience.

Only one in five primary school teachers (20.3%) stated positively that there were conditions in the school for the inclusion of children with disabilities. As the most common problems they had in the educational work with this category of students, the teachers of primary education stated the difficult adoption of the material (82.8%) and the realization of emotional control by 17.2%.

One in three teachers (32.8%) thought that inclusion was useful for children with disabilities (29.7%), of them thought that schools did not have the conditions for inclusive education, while one in four teachers (25.0%) believed that inclusion was not good. Every second professor (56.3%) thought that children with disabilities would be more successful in mastering the material in a special school, while 43.8% of them thought that they would be more successful in a regular school, but if there were schools that corresponded to material and organizational standards of the most developed countries. Observed by gender, the attitudes of teachers did not show a statistically significant difference, while according to work experience there are differences when it comes to attitudes toward inclusive education, to difficulties in working with children with disabilities and to the benefits of inclusive education (Table 1).

Despite positive attitudes toward inclusive education, teachers' attitudes were only partially positive when it came to attitudes toward children with disabilities (Table 2). Observed attitudes showed a statistically significant difference.

The professors also pointed out which factors would make it easier for a child with developmental disabilities to follow regular classes (Table 3) complete with the factors that most often complicated the inclusion process in practice (Table 4). Observed factors showed a statistically significant difference.

## Discussion

The significance of implementing inclusion in the education system complete with the association of early inclusive education with educational outcomes of children with disabilities is evident (Samadi and McConkey, 2018). Numerous countries have made considerable progress in this particular area, by means of legal regulation which regulates the provision of services to children with disabilities (Magyar et al., 2020). However, data obtained so far indicate that in some of the countries children with disabilities still attend special schools and are often excluded from the educational system. In order to enhance the inclusion system, local and national politics have to be precisely conceptualized with clearly defined objectives, whereas the school environment has to provide adequate support for children with disabilities (Werner et al., 2021).

Teachers are recognized as the key actors and their attitude toward inclusion is of high relevance for the successful implementation of inclusive education strategies, but the factors influencing these attitudes have been given insufficient attention in the studies conducted in our territories. Despite the fact that substantial action has already been taken in relation to this particular area, the implementation of inclusive education in practice has encountered numerous difficulties. It is our teachers who place an emphasis on the fact that inclusive education is an inalienable right of every child, they accentuate its usefulness in terms of understanding individual differences among children, but they also point out that regular schools have no conditions or capacities to carry out inclusive education.

Additionally, other research studies demonstrate that teachers justify the concept of inclusive education in terms of children's rights (Kayama, 2010; Okyere et al., 2019), but they also express their concern regarding numerous challenges they are currently juggling in their daily work with children with disabilities. The lack of professional competencies along with the lack of adequate conditions aimed at developing successful inclusive educational practice are the main reasons given by teachers which present a major hindrance to the successful implementation of inclusive education (Savic and Prosic-Santovac, 2017), which is indicated by our research findings.

Interestingly, the results obtained from several studies show that certain gender specific patterns are recognizable when it comes to attitudes of teachers toward inclusive education, with female teachers showing a more positive attitude toward inclusive education, whereas other studies indicated no gender specific differences in forming the aforementioned attitudes (Alghazo et al., 2003; Woodcock, 2013).

Concerning the age relevance, some studies demonstrate that the age of teachers has no significant effect on forming their attitudes toward inclusive education (Avramidis and Norwich, 2002), whereas other studies show that older teachers have more negative attitudes toward inclusion (De Boer et al., 2011). In addition, they indicate that inclusive education training programs have a positive effect on attitudes of younger teachers, which is probably due to the fact that older teachers have rarely attended courses on inclusive teaching or have not been provided with relevant in-service training at all (Forlin et al., 2009). Our research results show that sex have no significant impact on their attitudes toward inclusive education.

Another study outlined that the lack of teachers' self-esteem regarding teaching students with disabilities was associated with their forming negative attitudes toward inclusion (Avramidis et al., 2020), whereas the study which took into consideration the factors such as the type and size of the school building and its classrooms complete with the characteristics of students such as gender or whether the child received support in the academic or non-academic areas of school education—indicated that the aforementioned factors had no significant effects on the attitudes of teachers toward inclusive education (Vaz et al., 2015).

Our study showed that teachers reported that the inclusion process could be more easily facilitated by the classroom adaptations accompanied by a class size reduction. However, we did not take into consideration the characteristics of students with disabilities in terms of their gender and level of school achievement in the inclusive environment, which may be the subject of our future research.

Taking into account the fact that the teachers included in our study reported insufficient curriculum planning for teaching children with disabilities, it is more than evident how necessary it is to introduce special curriculum programs for inclusion education. They emphasize that reforms of excessive coursework and curricula should be delivered, along with the adaptation of teaching contents to the abilities of these children and the application of active, interactive and participatory teaching methods. In addition, they indicated the necessity of continuous provision of professional assistance to the teacher by special educators/experts, such as defectologists and social pedagogues.

A large number of research evidence speak out in favor of the fact that it was the provision of support to teachers that positively influenced their attitudes toward inclusion, indicating that after the completion of inclusion training programs—the level of positive attitudes of teachers toward inclusion was significantly raised. Additionally, they emphasized that various types of social support (such as informational, instrumental or emotional support) may be provided by various actors (colleagues, supervisors, etc.) (Desombre et al., 2021; Hassanein et al., Hassanein et al.).

Nevertheless, the findings of other studies revealed that in the case if children or adults with disabilities were among teachers' family members or their circles of friends, teachers were more open to embracing the concept of inclusion, and that the very knowledge they possessed on the specific disability of their students had a positive impact on their attitudes toward inclusion (Vaz et al., 2015).

Teachers have to actively participate in the process of inclusion implementation and they have to be ready to handle all the new challenges presented at each level of the education system. They primarily need adequate support, cooperation, provision of professional experience and specific preparation of individual class sessions for the purpose of achieving as high-quality level of inclusive education as possible. Multidisciplinary work, professional development, attitudes and perception of teachers along with adequate cooperation with their students' family members and provision of positive support to students—play a significant role in inclusive education (Rojo-Ramos et al., 2021).

The inclusion of special needs children presents one of a kind challenge for the societies worldwide. Therefore, we need to take concrete actions and establish organizations of such a kind in all societies for the purpose of implementing adequate and high-quality inclusive education for this group of children specifically. Educational institutions should transform and adjust their educational programs in order to respond to this new growing challenge, whereas teachers should use their positive attitudes toward inclusive education to assume the leading role (Tétreault et al., 2014).

The advantages of our particular study are related to providing insight into the factors perceived as positively or negatively affecting the formation of positive attitudes toward inclusive education. It can contribute to creating measures, the focus of which will be on the enhancement and development of positive attitudes of teachers toward inclusive education. It is more than clear that there is a number of mutually linked factors and that each of the factors should be given considerable attention and be subjected to a detailed analysis. However, our study encountered certain limitations of its own. Namely, the sample included the teachers from urban areas only, whereas the teachers from neighboring suburban and rural areas were not included in the study. Additionally, the following factors were not included in the study: general attitudes of teachers toward people with disabilities in the society as a whole, and possible culturological differences between them. Furthermore, we did not take into consideration the degree of their concern or stress caused by the actual physical contact with children with disabilities, nor did we consider the financial fees for their work. We did not consider the attitudes of students and their parents toward inclusive education, investigations in this particular area will be a significant component of the future research studies, especially because of the fact that the teachers included in our study had already emphasized that the following factors complicated the process of inclusion in education: resistance of other children toward children with disabilities and resistance of parents of children of typical development toward inclusion. All the aforementioned facts indicated the necessity and recommendations for our future research directions. Due to our current study, numerous questions have been raised which are going to be part of our future research endeavors.

## Conclusions

In order to guarantee the smooth inclusion of children with disabilities, various studies of this type are needed to identify the factors that hinder inclusive education in order to formulate strategies that can improve the inclusion of children with intellectual and developmental disabilities in the education system. Developing policies that support such strategies could improve this implementation. Experts specialized in different fields should gather in order to identify practical solutions to the challenges of creating an inclusive environment for children with special needs. Investing more resources and time in developing and implementing special education policies can promote successful inclusive education. The implementation of inclusive education is a very complex process and if it is considered from different perspectives, we could create a possibility of using the results of all the research conducted so far as well as all the future research results as the basis for initiating national programs and strategies on inclusive education. Our study represents only the starting point of our aspirations to render the inclusive education process in our territories as high-quality and comprehensive as possible.

## Data Availability Statement

The original contributions presented in the study are included in the article/supplementary material, further inquiries can be directed to the corresponding author.

## Author Contributions

JR conceptualized the research. JR, TK, SRade, and MM conducted the literature review, a preliminary analysis of the data, and a first draft of the manuscript. SRado and MS revised the data analysis. KP, MD, SC, BR, and OD revised the manuscript and provided feedback and corrections. JN, IS, KJ, and JR revised the final version of the manuscript. All authors contributed to the article and approved the submitted version.

## Conflict of Interest

The authors declare that the research was conducted in the absence of any commercial or financial relationships that could be construed as a potential conflict of interest.

## Publisher's Note

All claims expressed in this article are solely those of the authors and do not necessarily represent those of their affiliated organizations, or those of the publisher, the editors and the reviewers. Any product that may be evaluated in this article, or claim that may be made by its manufacturer, is not guaranteed or endorsed by the publisher.
